# APOE4 differentially alters lipid composition in extracellular vesicles from hiPSC-derived neurons of Alzheimer’s disease patients

**DOI:** 10.3389/fphar.2026.1917428

**Published:** 2026-09-25

**Authors:** Marios G. Krokidis, Ram Sagar, Maja Mustapic, Maria-Christina Papatheodorou, Themis P. Exarchos, Constantine G. Lyketsos, Panagiotis Vlamos, Dimitrios Kapogiannis, Vasiliki Mahairaki

**Affiliations:** 1 Bioinformatics and Human Electrophysiology Laboratory, Department of Informatics, Ionian University, Corfu, Greece; 2 Institute of Digital Biomedicine, Ionian University Research and Innovation Center, Corfu, Greece; 3 Department of Genetic Medicine, Johns Hopkins University School of Medicine, Baltimore, MD, United States; 4 The Richman Family Precision Medicine Center of Excellence in Alzheimer’s Disease, Johns Hopkins University School of Medicine, Baltimore, MD, United States; 5 Laboratory of Clinical Investigation, National Institute on Aging, National Institutes of Health, Baltimore, MD, United States; 6 Department of Psychiatry and Behavioral Sciences, Johns Hopkins University School of Medicine and Johns Hopkins Bayview Medical Center, Baltimore, MD, United States

**Keywords:** Alzheimer’s disease, extracellular vesicles, induced pluripotent stem cells, lipid profile, neurodegenerative diseases, plasma biomarkers

## Abstract

**Background:**

The Apolipoprotein E4 (APOE4) is the most significant genetic risk factor for late-onset Alzheimer’s disease (AD), but the cellular mechanisms by which it increases vulnerability remain poorly understood. Lipids play a crucial role in membrane organization, vesicle trafficking, and inflammatory signaling in the brain. Increasing evidence suggests that changes in lipid metabolism might serve as a connection between genetic risk and neurodegeneration. Extracellular vesicles (EVs) reflect the lipidomic composition of their parent cell offering a window into lipidomic alterations that may be tracked in living humans over time. Given high variability in human samples, we sought to define APOE4-related lipidomic EV signatures in a controlled human cellular system.

**Methods:**

We studied EVs secreted by four replicates of human-induced pluripotent stem cell (iPSC)-derived glutamatergic neurons from two healthy individuals (APOE2/E3 and APOE4/4) and two patients with AD (APOE3/3 and APOE4/4). Targeted lipidomics enabled quantitative profiling of hundreds of individual lipid species across major lipid classes. Univariate and multivariate analyses were applied to identify lipid alterations associated with APOE genotype and AD status.

**Results:**

APOE4 was associated with differences in lipid composition in neuronal EVs from both healthy and AD donors, suggesting that APOE4 influences EV lipid composition regardless of AD. Additionally, APOE4/4 carriers with AD exhibited clear, species-specific lipid imbalances. Lower levels of ganglioside GM3 and higher of phosphatidylserine emerged as consistent contributors to the APOE4-associated AD lipid signature, alongside alterations in dihydrosphingomyelin and phosphatidylinositol species linked to membrane integrity, endolysosomal function, and inflammatory pathways. These lipid species were among the strongest variables distinguishing healthy and AD APOE4/4 samples in multivariate analyses.

**Conclusion:**

These findings specify lipidomic changes in AD related to the APOE4 genotype, and support a model by which APOE4 creates a lipid profile that becomes selectively dysregulated in AD. By identifying specific lipid species associated with APOE4 and AD, this work provides further evidence that distinct lipidomic signatures are associated with both APOE4 and AD. These findings may contribute to the identification of lipid-based biomarkers and support future investigations into genotype-informed therapeutic strategies, although validation in larger, independent cohorts is required.

## 1 Introduction

Alzheimer’s disease (AD) is a progressive neurodegenerative disorder characterized by the gradual deterioration of cognitive function and, ultimately, the capacity to perform basic daily activities. The condition predominantly affects older adults and represents the most common cause of dementia worldwide (Tenchov et al., 2024). Apolipoprotein E (APOE) is the strongest genetic factor associated with late-onset sporadic AD. Its three common alleles (ε2, ε3, and ε4) shape disease risk along a continuum, with ε4 conferring higher susceptibility and earlier disease onset compared with other APOE isoforms (Di Battista et al., 2016) with ε2 offering relative protection (Raulin et al., 2022). Notably, APOE-related risk varies across populations due to differences in genetic ancestry and environmental influences (Abondio et al., 2019). In terms of underlying mechanisms, APOE was initially linked to AD pathogenesis through its effects on amyloid-β (Aβ) clearance (Chen et al., 2025). However, it is now understood that APOE participates in a wide array of pathogenic processes, including tau phosphorylation, neuroinflammation, as well as susceptibility to lipid peroxidation and ferroptosis (Belaidi et al., 2025). The ε4 isoform negatively influences many of these pathways, though its exact molecular actions remain only partially defined.

In recent years, lipidomics have emerged as a powerful approach for investigating metabolic alterations in biological samples from patients with AD (Wretlind et al., 2024). Lipids play critical roles in membrane structure, cellular signaling and energy metabolism across cellular compartments. Lipid species distribution may reflect early disruptions in brain function and may be influenced by both genetic background and other AD pathologies (Chew et al., 2020). In one study, AD patients exhibited a distinct plasma lipid profile compared with age-matched healthy individuals, with notably higher levels of phosphatidylcholines (PC) (Liu et al., 2021). In another, targeted plasma lipidomics revealed significant differences in diglycerides and sphingomyelins in preclinical and MCI-AD groups, with 18:1 LPE showing higher plasma levels compared with cognitively unimpaired controls (Peña-Bautista et al., 2022). However, these studies examined lipidomic signatures in plasma cannot distinguish peripheral from brain-specific findings.

In recent years, the development of techniques that isolate extracellular vesicles (EVs) derived from neurons and other brain cells has allowed the study of brain-specific lipidomic signatures in living cells. Encircled by a phospholipid bilayer membrane enriched in cholesterol and ceramides, EVs transport proteins, lipids, and nucleic acids between cells, thereby playing essential roles in cellular homeostasis, intercellular communication, and the regulation of various physiological and pathological processes (Morelli, 2017; Hallal et al., 2022). Cell-type-specific EVs exert multifaceted effects in the development and progression of AD, contributing to amyloid-β (Aβ) production and oligomerization, while also promoting the clearance of toxic protein aggregates (Gomes et al., 2022). These diverse and context-dependent EV functions emphasize both neuroprotective potential and contributions to understanding AD pathophysiology. Neuronal-origin EVs in blood provide a more sensitive and specific platform for mechanistic discovery than bulk plasma or serum, enabling the detection of low-abundance biomarkers, including phosphorylated tau (p-tau), that may otherwise remain undetectable in circulating plasma (Mustapic et al., 2017). We recently reported distinct differences in the levels of selected species of glycerolipids, glycerophospholipids, lysophospholipids and sphingolipids across three distinct EV subpopulations (neuronal, astrocytic and systemic) from plasma samples of individuals with AD or cognitively unimpaired controls (Krokidis et al., 2024). However, to properly interpret biomarker studies and given high variability in human plasma samples, clear signatures related to underlying genetic factors and the presence of disease are needed. Such signatures require using controlled human cellular models.

Human induced pluripotent stem cell (iPSC) technologies have transformed preclinical research, enabling researchers to generate patient-specific human cell types *in vitro* and apply them to the study of a wide range of conditions, including neurodegenerative diseases (Penney et al., 2020). Over the past 2 decades, iPSC-based models have become especially valuable in AD, where disease-modifying treatments remain limited. By comparing iPSC-derived brain cells from patients with AD and healthy controls, researchers have identified variability in cellular phenotypes across diverse genetic backgrounds (Barak et al., 2022). Combined with precise genome-engineering tools that allow the introduction or correction of AD-associated variants, patient-derived iPSCs now provide powerful platforms for uncovering AD pathology and advancing therapeutic discovery (Mahairaki et al., 2011; Sullivan and Young-Pearse, 2017; Penney et al., 2020).

Extracellular vesicles (EVs) derived from iPSC-differentiated glutamatergic neurons provide a controlled, cell-type-specific system that reflects the neuronal lipid landscape directly relevant to AD pathophysiology, circumventing the high inter-individual variability and peripheral contamination inherent in human plasma samples. Furthermore, by isolating EVs from iPSC-derived neurons generated from donors with defined APOE genotypes (APOE2/3, APOE4/4) and distinct disease status (AD vs. unimpaired), this approach enables the precise dissection of lipid alterations attributable to APOE4 genotype from those driven by AD pathology itself, a resolution not achievable through bulk plasma lipidomics alone. Herein, human peripheral blood mononuclear cells (PBMCs) were collected from 2 unimpaired individuals and 2 patients with AD, each stratified by APOE genotype (specifically, healthy APOE2/E3, healthy APOE4/4, AD APOE3/3 and AD APOE4/4). These cells were reprogrammed into iPSCs, differentiated into glutamatergic neurons, and subsequently, EVs were isolated in culture. Targeted lipidomic analysis was conducted to identify alterations in lipid composition across clinically and genetically distinct groups. The design allowed us to distinguish lipid changes driven by APOE4 from those caused by AD and to examine how each shape EVs lipid composition. Together, this approach helps identify lipid changes linked to genetic risk and disease state and clarifies how APOE4 contributes to AD pathogenesis.

## 2 Materials and methods

### 2.1 Collection of human PBMCs

Blood from unimpaired APOE2/E3, unimpaired APOE4/4, AD APOE3/3 and AD APOE4/4 patients was collected through the Johns Hopkins Alzheimer’s Disease Research Center (ADRC). The characteristics of study participants have been reported (Sagar et al., 2023a; Sagar et al., 2023b). Study oversight was provided by a Johns Hopkins Institutional Review Board (JHIRB). Informed consent was obtained from all participants before data and sample collection.

### 2.2 Human iPSC generation and neuronal differentiation

Human iPSCs were generated from PBMCs by transient expression of the plasmid vectors (MOS, MMK, and GBX) using a 4D Nucleofector under serum-free culture conditions. The hiPSC lines were thoroughly characterized by immunocytochemistry for the pluripotency markers (TRA-1-60, NANOG, and OCT4), flow cytometry (TRA-1-60), karyotyping to confirm chromosomal normality, and their capacity to differentiate into all three germ layers. The hiPSC cultures were also randomly screened for *mycoplasma* contamination, and all tested samples were negative. For neuronal differentiation, all iPSC lines were transduced with lentivirus (expressing NgN2 and rtTA) as described in a previously established protocol to acquire a pure and high population of excitatory neurons within 2 weeks (Sagar et al., 2023a; Sagar et al., 2023b). For neuronal differentiation, NgN2-positive cells were seeded in a vitronectin-coated plate at a density of 250,000 cells/well in E8 medium supplemented with ROCK inhibitor. The cells were maintained in E8 medium until they reached 90% confluency, and then doxycycline was added to induce Ngn2 expression (Day 0). On Day 1, the cells were selected using puromycin in the induction medium. The next day, cells were passaged onto Matrigel-coated (6-well) plates. On Day 4, the media (50%) was replaced with Neuronal maturation media supplemented with Ara-C to inhibit the proliferation of non-neuronal cell types in the culture. From Day 6 to Day 12, neuronal maturation media (70%) were changed on alternate days. From Day 15 to Day 27, the maturation media (50%) without doxycycline were changed every 48 h. On Day 27, mature neuronal cells were collected for EV isolation, characterization, and targeted lipidomic analysis.

### 2.3 Extracellular vesicle isolation

EVs were isolated from the cell culture medium of the iPSC-derived neurons of the AD and unimpaired controls [4 biological replicates x 4 cell lines (healthy APOE2/E3, healthy APOE4/4, AD APOE3/3 and AD APOE4/4)] using qEV 70 nm size exclusion chromatography (SEC) with qEV columns as previously described (Sagar et al., 2025). On day 27 of culture, 12 mL of cell supernatant were collected, centrifuged at 300 g for 10 min at 4 °C, and further centrifuged at 2000 g for 10 min at 4 °C to remove cell debris. Then, the supernatant was concentrated to 500 μL using an Amicon Ultra-15 centrifugal filter unit (Ultracel-100KD) at 2000 g for 20 min. The concentrated sample was loaded onto a qEV-70 SEC-column and eluted with DPBS. The first 2 mL of EV-containing fractions were collected and concentrated again to 200 μL using an Amicon Ultra-2 centrifugal filter unit (Ultracel-10KD). This workflow was previously evaluated and optimized using early EV-enriched fractions, late protein-enriched fractions, and buffer-only controls (Sagar et al., 2025). The resulting EV-enriched particles were characterized by NanoFCM, TEM, and SP-IRIS and used for lipidomic analysis; therefore, the lipidomic profiles represent the combined isolated EV population rather than an individual EV subtype.

### 2.4 Nano-flow cytometry measurements (NanoFCM) analysis

The concentration and size distribution of EVs were measured using the Flow Nano Analyzer (NanoFCM) according to the manufacturer’s guidelines and established protocols (Huang et al., 2023; Arab et al., 2021). EV samples were diluted in DPBS, and recordings were made for 1 minute. Particle numbers were calculated from a calibration curve based on flow rate and side scatter intensity.

### 2.5 Transmission electron microscopy (TEM)

EVs (10 µL) were imaged by TEM using established protocols (Huang et al., 2020; Arab et al., 2021). The samples were applied to copper grids, washed with Tris-buffered saline, and stained with 1% uranyl acetate mixed with tylose. After blotting, the grids were imaged at 60,000× magnification on a Hitachi 7600 TEM at 80 kV and analyzed with ImageJ software, in which all particle structures were manually selected and measured for quantification.

### 2.6 Single particle interferometric reflectance imaging (SP-IRIS)

The EV phenotype was assessed using the Single-Particle Interferometric Reflectance Imaging (SP-IRIS) procedure as previously described (Huang et al., 2020; Huang et al., 2023; Arab et al., 2021). EVs were diluted to 1.51E + 9 particles/mL using an incubation solution from a Leprechaun human tetraspanin kit, and 40 µL of this preparation was incubated on the chip surface. After incubation, the chips were washed and imaged with an ExoView R100 instrument, and the data were analyzed using NanoViewer 3.0.14 software.

### 2.7 Targeted lipidomics

Analysis of 16 EV samples (4 biological replicates × 4 cell lines) was conducted at the Biomarkers Core Laboratory (BCL) of Columbia University. Briefly, lipidomic profiling was performed using Ultra Performance Liquid Chromatography-Tandem Mass Spectrometry (UPLC-MSMS) (Agudelo et al., 2020; Area-Gomez et al., 2021). Lipid extracts were prepared from samples spiked with appropriate internal standards using a modified Bligh and Dyer (Bligh and Dyer, 1959) method and analyzed on a platform comprising Agilent 1260 Infinity HPLC integrated to Agilent 6490A QQQ mass spectrometer controlled by Masshunter v 7.0 (Agilent Technologies, Santa Clara, CA). Glycerophospholipids and sphingolipids were separated with normal-phase HPLC as described before (Chan et al., 2012), with a few modifications. An Agilent Zorbax Rx-Sil column (2.1 × 100 mm, 1.8 µm) maintained at 25 °C was used under the following conditions: mobile phase A (chloroform: methanol: ammonium hydroxide, 89.9:10:0.1, v/v) and mobile phase B (chloroform: methanol: water: ammonium hydroxide, 55:39:5.9:0.1, v/v); 95% A for 2 min, decreased linearly to 30% A over 18 min and further decreased to 25% A over 3 min, before returning to 95% over 2 min and held for 6 min. Separation of sterols and glycerolipids was carried out on a reverse phase Agilent Zorbax Eclipse XDB-C18 column (4.6 × 100 mm, 3.5um) using an isocratic mobile phase, chloroform, methanol, 0.1 M ammonium acetate (25:25:1) at a flow rate of 300 μL/min. Quantification of lipid species was accomplished using multiple reaction monitoring (MRM) transitions (Chan et al., 2012),] under both positive and negative ionization modes in conjunction with referencing of appropriate internal standards: PA 14:0/14:0, PC 14:0/14:0, PE 14:0/14:0, PG 15:0/15:0, PI 17:0/20:4, PS 14:0/14:0, BMP 14:0/14:0, APG 14:0/14:0, LPC 17:0, LPE 14:0, LPI 13:0, Cer d18:1/17:0, SM d18:1/12:0, dhSM d18:0/12:0, GalCer d18:1/12:0, GluCer d18:1/12:0, LacCer d18:1/12:0, D7-cholesterol, CE 17:0, MG 17:0, 4ME 16:0 diether DG, D5-TG 16:0/18:0/16:0 (Avanti Polar Lipids, Alabaster, AL). Lipid levels for each sample were calculated by summing up the total number of moles of all lipid species measured across all three LC-MS methodologies and then normalizing that total to mol%.

### 2.8 Data preprocessing

The dataset comprises comprehensive quantitative lipidomic profiles derived from 16 EV samples: 4 biological replicates of 4 cell lines, i.e., healthy APOE4/4, healthy APOE2/E3, AD APOE4/4, and AD APOE3/3. The measurements encompass both aggregate lipid classes and a detailed resolution of individual lipid species within each class. Raw data were imported from structured excel spreadsheets and preprocessed in R. Numeric values corresponding to lipid classes concentrations were isolated and lipid identifiers were retained as row labels.

### 2.9 Statistical analysis and feature selection

We defined genotype groups based on APOE4 (i.e., unimpaired APOE4/4 and AD APOE4/4 vs. unimpaired APOE2/E3 and AD APOE3/3) and disease based on presence or absence of AD (i.e., unimpaired APOE4/4 and unimpaired APOE2/E3 vs. AD APOE4/4 and AD APOE3/3). The data were expressed as the mean value ± standard deviation (SD) for lipids, based on measurements from two samples per group. Statistical significance was assessed by unpaired two-tailed Student's t-tests. In addition to this main analysis, we performed a multivariate analysis to elaborate on univariate findings. To assess the statistical significance of group-level differences, pairwise comparisons were conducted for each lipid species using either parametric or non-parametric tests, depending on the data distribution. The resulting *p*-values were used to identify a preliminary set of candidate lipids that may exhibit biologically meaningful differences between groups. Principal component analysis (PCA) was performed on a centered and scaled data matrix to explore the overall structure and variability of the dataset. Volcano plots were generated for each group comparison to simultaneously visualize statistical significance (–log10 *p*-value) and magnitude of change (log2 fold-change). To control for multiple hypothesis testing and reduce the risk of type I errors, *p*-values from the univariate statistical tests were adjusted using the Benjamini–Hochberg false discovery rate (FDR) procedure implemented in R. Lipids with an FDR-adjusted *p*-value below 0.05 were selected as significant. This approach reduces the proportion of false positives, ensuring that retained lipids are more likely to reflect genuine biological effects and can be confidently used in subsequent analyses.

## 3 Results

Human iPSC derived neuronal EVs were isolated from the conditioned medium using a SEC column-based method combined with ultrafiltration washes and conducted targeted lipidomic analysis post through characterization using NanoFCM, TEM, and SP-IRIS as shown in the study flow diagram (Figure 1A). Our results indicate successful isolation of EVs. NanoFCM revealed similar size distributions of EVs for both AD and control samples (Figures 1B,C). Representative TEM confirmed similar-sized EVs in both AD and control samples (Figure 1D). SP-IRIS detected typical EV tetraspanin markers CD63, CD81, and CD9 in the captured population with no significant difference in mean EV diameter between AD and unimpaired controls. We observed higher detection rates for CD63 and CD81 compared to CD9, along with co-localization of CD63 and CD81 (Figure 1E). Size profile of tetraspanin-captured with no significant difference between all AD and unimpaired controls using SP-IRIS (Figure 1F).

**FIGURE 1:**
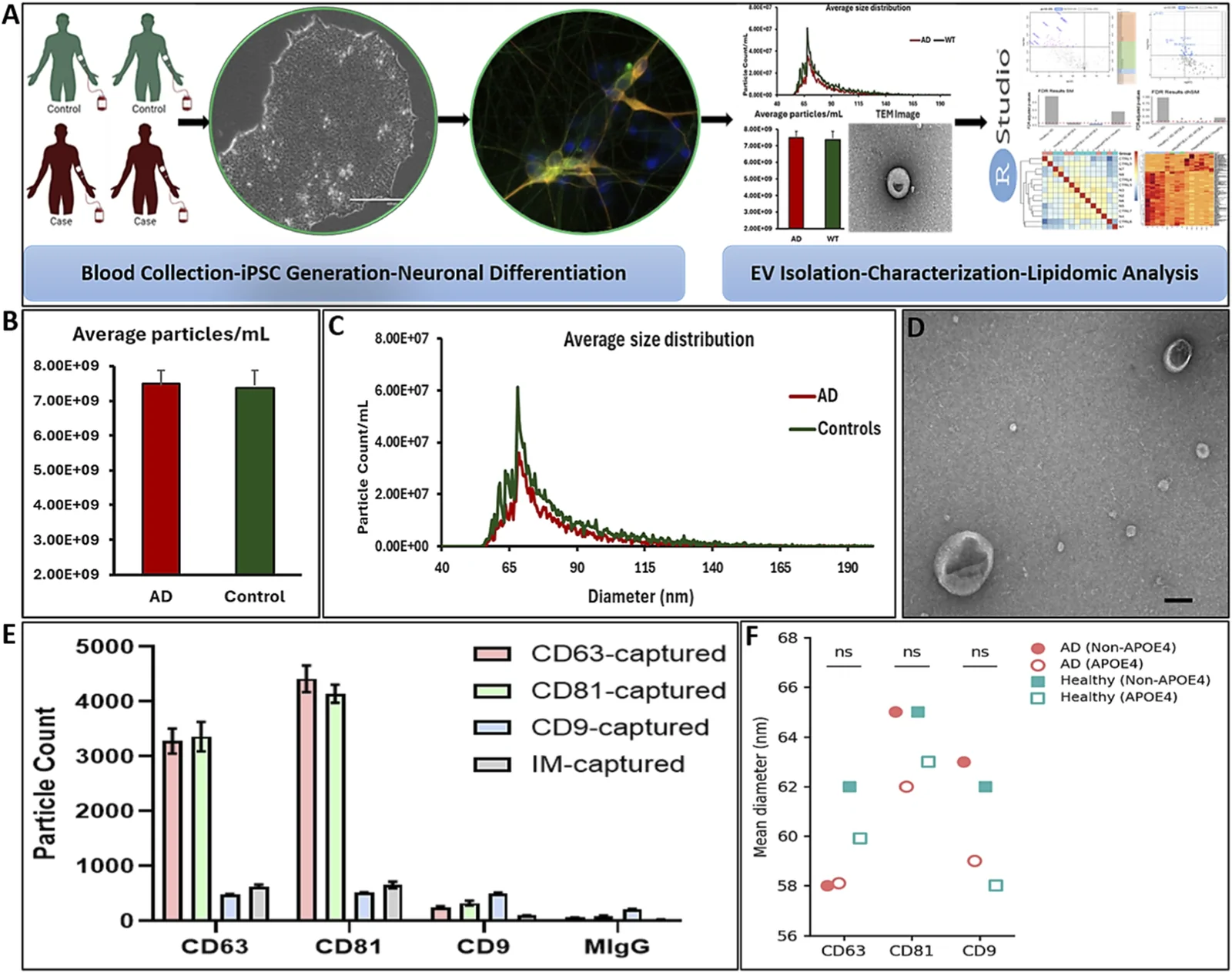
Study workflow and neuronal EV characterization. **(A)** Study flow diagram from blood collection, HiPSC generation, neuronal differentiation, and EV isolation, characterization, and lipidomic analysis. **(B)** Quantification of EV concentration (particles/mL) in AD and control samples measured by nano-flow cytometry (NanoFCM). **(C)** Average size (diameter) distribution analysis by NanoFCM shows that both (AD & control) samples were enriched in EV particles with a peak size around ∼70 nm. **(D)** Representative negative staining by transmission electron microscopy (TEM) to visualize EVs (scale bar-100 nm). **(E)** The presence and colocalization of tetraspanins CD63, CD81, and CD9 on EVs were analyzed by SP-IRIS. **(F)** Size profile of tetraspanin-captured AD and control samples using SP-IRIS.

Targeted lipidomic analysis was performed on 16 EV samples (4 donors, 4 biological replicates per donor; for each donor-derived cell line, 4 independent neuronal cultures were derived and were subjected to independent EV isolation and lipidomic analysis as biological replicates), allowing the quantification of 593 lipid species, including lipid classes from the sterol lipid, glycerolipid, sphingolipid, glycerophospholipid and lysophospholipid categories. The relative composition of EVs in the identified lipid categories expressed as normalized relative abundance differed across disease and genotype groups, as shown in Figure 2 (also Supplementary Tables S1,S2). Significant reduction in sterol lipids and in sphingolipids (p = 0.033 and p = 0.049, respectively) was observed in the unimpaired APOE4/4 person compared to the unimpaired person APOE2/E3 (Figures 2A,C, respectively). Furthermore, lower levels of glycerophospholipids pool (Figure 2D) were noted in unimpaired APOE4/4 to unimpaired APOE2/E3 (p = 0.044) as well as between AD APOE4/4 and unimpaired APOE4/4 (p = 0.0479), while significant enhancement was found in lysophospholipid abundance in AD APOE3/3 compared to unimpaired APOE2/E3 (p = 0.028) (Figure 2E).

**FIGURE 2:**
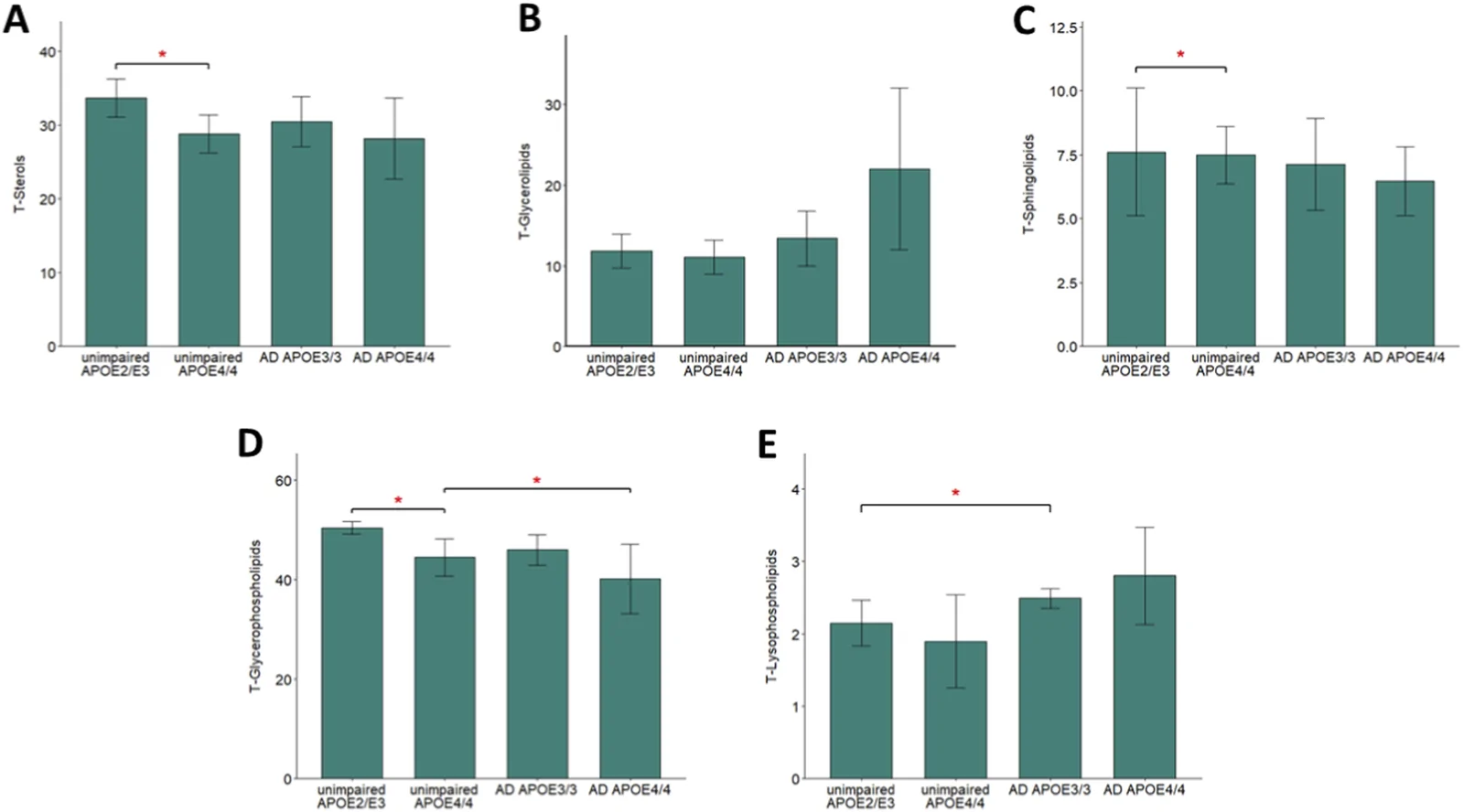
Relative quantitative percentage of the average of main lipid categories obtained from distinct groups of EVs from hiPSC-derived neurons of unimpaired control and AD samples (both APOE2/E3 and APOE4/4). **(A)** sterol lipids, **(B)** glycerolipids, **(C)** sphingolipids, **(D)** glycerophosholipids and **(E)** lysophosholipids. The values are given as mean ± SD (n = 4). Statistical significances: *(p < 0.05). For specific values see Supplementary Table S1.

Comparing specific lipid classes, substantial differences were detected (Figure 3; Supplementary Table S3 for details). Among sterol lipids, free cholesterol was significantly decreased in healthy APOE4/4 compared to APOE2/E3 (p = 0.0449) (Supplementary Figure S1). No differences were observed in monoglycerides, diglycerides or triglycerides counterparts between the groups (Supplementary Table S4). Focusing on sphingolipids, elevated levels of dihydrosphingomyelin (dhSM) were obtained between unimpaired APOE4/4 and unimpaired APOE2/E3 (p = 0.4845), whereas a diminution in dhSM was observed in AD APOE4/4 samples compared with unimpaired APOE4/4 (p = 0.0135) (Figure 3A). Higher levels of monosialodihexosylganglioside were found in unimpaired APOE4/4 compared to unimpaired APOE2/E3 (p = 0.01207), while lower levels were detected in AD APOE4/4 compared to AD APOE3/3 (p = 0.03968) as well as to healthy APOE4/4 (p = 0.01140) (Figure 3B). Lastly, globotriaosylceramide was increased in AD APOE3/3 compared to unimpaired APOE2/E3 (p = 0.03291), on the other hand, levels were lower compared to AD APOE4/4 (p = 0.04366) (Figure 3C).

**FIGURE 3:**
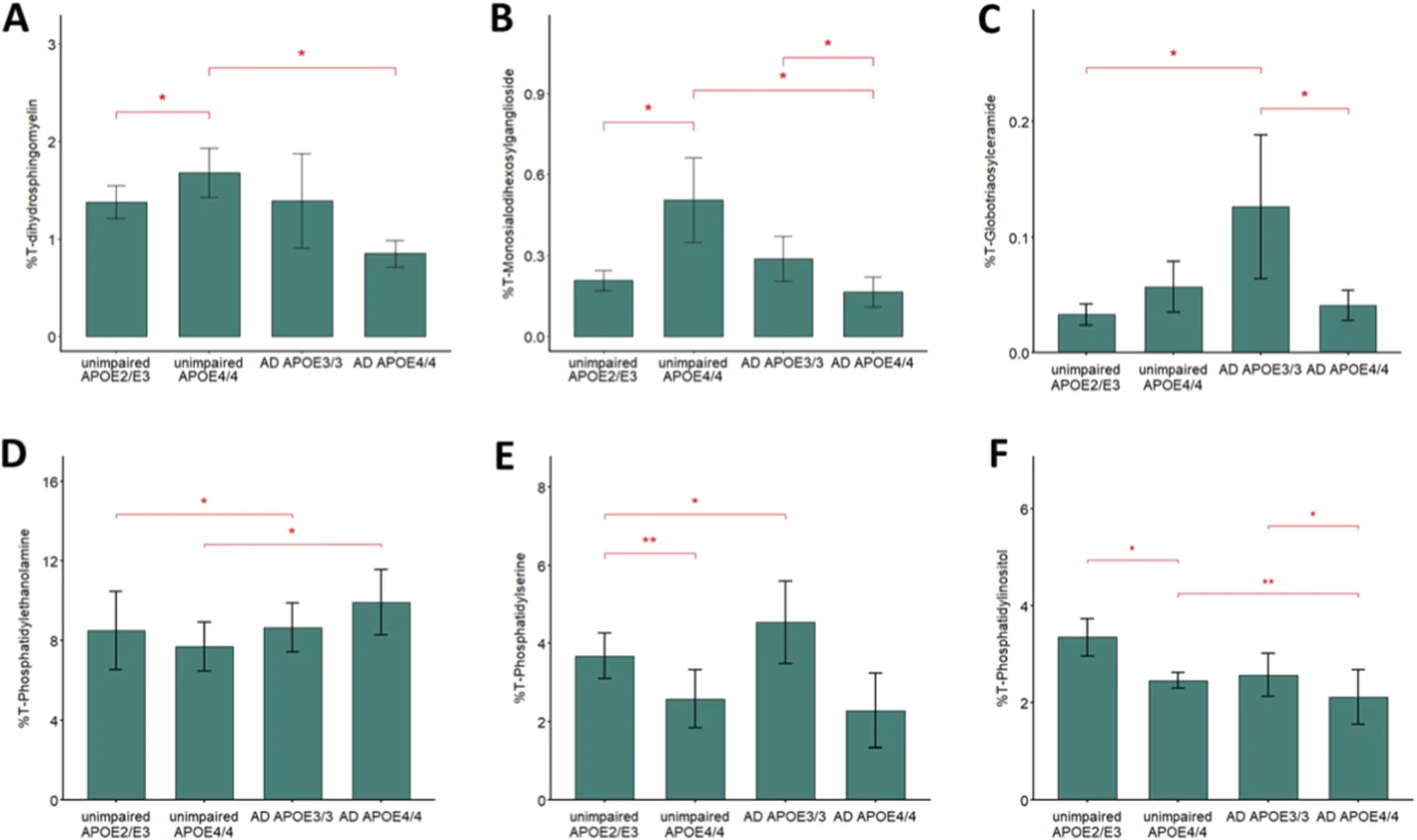
Relative quantitative percentage of representative classes of sphingolipids (SLs) glycerophospholipids (GPLs) and lysophospholipids (LPLs) in EVs from hiPSC-derived neurons of healthy control and AD samples (both APOE2/E3 and APOE4/4). **(Α)** Dihydrosphingomyelin (dhSM), **(Β)** Monosialodihexosylganglioside (GM3), **(C)** Globotriaosylceramide (GB3), **(D)** Phosphatidylethanolamine (PE), **(E)** Phosphatidylserine (PS), **(F)** Phosphatidylinositol (PI). The values are given as mean ± SD (n = 4). Statistical significance: *(p < 0.05), **(p < 0.05).

Given the limited number of independent donors, these findings should be considered exploratory. Within this context, analysis of glycerophospholipids revealed lower levels of phosphatidylcholine (PC) for AD APOE4/4 compared to unimpaired APOE4/4 samples (p = 0.0495). Significantly elevated levels of phosphatidylethanolamine (PE) were also observed comparing AD APOE3/3 and unimpaired APOE2/E3 (p = 0.0195) as well as between AD APOE4/4 and unimpaired APOE4/4 (p = 0.0089) (Figure 3D). On the other hand, phosphatidylserine was lower in unimpaired APOE4/4 and higher in AD APOE3/3 compared to unimpaired APOE2/E3 (p = 0.0043 and p = 0.0374, respectively), as Figure 3E highlights. For phosphatidylinositol, lower levels were noted in unimpaired APOE4/4 compared to unimpaired nonAPOE4/4 (p = 0.0139) and in AD APOE4/4 compared to AD nonAPOE4/4 (p = 0.0347) as well as between AD APOE4/4 and unimpaired APOE4/4 samples (p = 0.0082) (Figure 3F). Lysophospholipids, deacylated forms of phospholipids with a single fatty acid chain, were also measured with an enhancement of lysophosphatidyserine in AD APOE3/3 unimpaired to healthy APOE2/E3 (p = 0.0213) with lower levels in AD APOE4/4 (p = 0.0110) (Supplementary Figure S1).

To evaluate further the metabolic effects of the APOE4 genotype and its interaction with AD pathology, Principal Component Analysis (PCA) was performed on the lead lipidomic classes (Figure 4). These findings suggest that APOE4 status may be associated with distinct lipidomic alterations that are detectable independently of AD status. Specifically, for dihydrosphingomyelins (dhSM), unimpaired APOE4/4 carriers exhibited high metabolic rigidity, with PC1 accounting for 98.2% of the variance and showing tighter clustering than non-carriers (Figure 4A). This stability was significantly disrupted in the AD APOE4/4 group, where pathology induced a directional shift in the lipidomic profile (Figure 4B). The most pronounced genotype-dependent dysregulation was observed in ganglioside GM3 levels. Samples from unimpaired APOE4/4 carriers displayed substantial variance dispersion (PC1: 94.5%) compared to samples from non-carriers, suggesting a loss of homeostatic control (Figure 4C). Notably, GM3 showed the most evident separation pattern between healthy and AD samples within the APOE4 background. The observed variation reflected a multidimensional pattern, with PC1 explaining 28.5% of the total variance and PC2 explaining 19.7%. The PCA plot revealed a tendency toward separation between the healthy and AD groups. To further assess the robustness of the observed group separations, t-distributed stochastic neighbor embedding (t-SNE) analysis was performed. The resulting visualizations largely confirmed the separation patterns indicated by PCA. Specifically, comparisons involving dihydrosphingomyelin, phosphatidylserine and phosphatidylinositol showed distinct, well-separated clusters in the t-SNE projections, closely mirroring the corresponding PCA results (Supplementary Figure S4). In contrast, monosialoganglioside and lysophosphatidylserine exhibited only partial separation, with the two groups occupying overlapping regions in the t-SNE projection (Supplementary Figures S2,S5). These findings suggest that the separation observed by PCA for these lipid classes should be interpreted with caution, as it was not consistently reproduced by this complementary non-linear dimensionality reduction approach.

**FIGURE 4:**
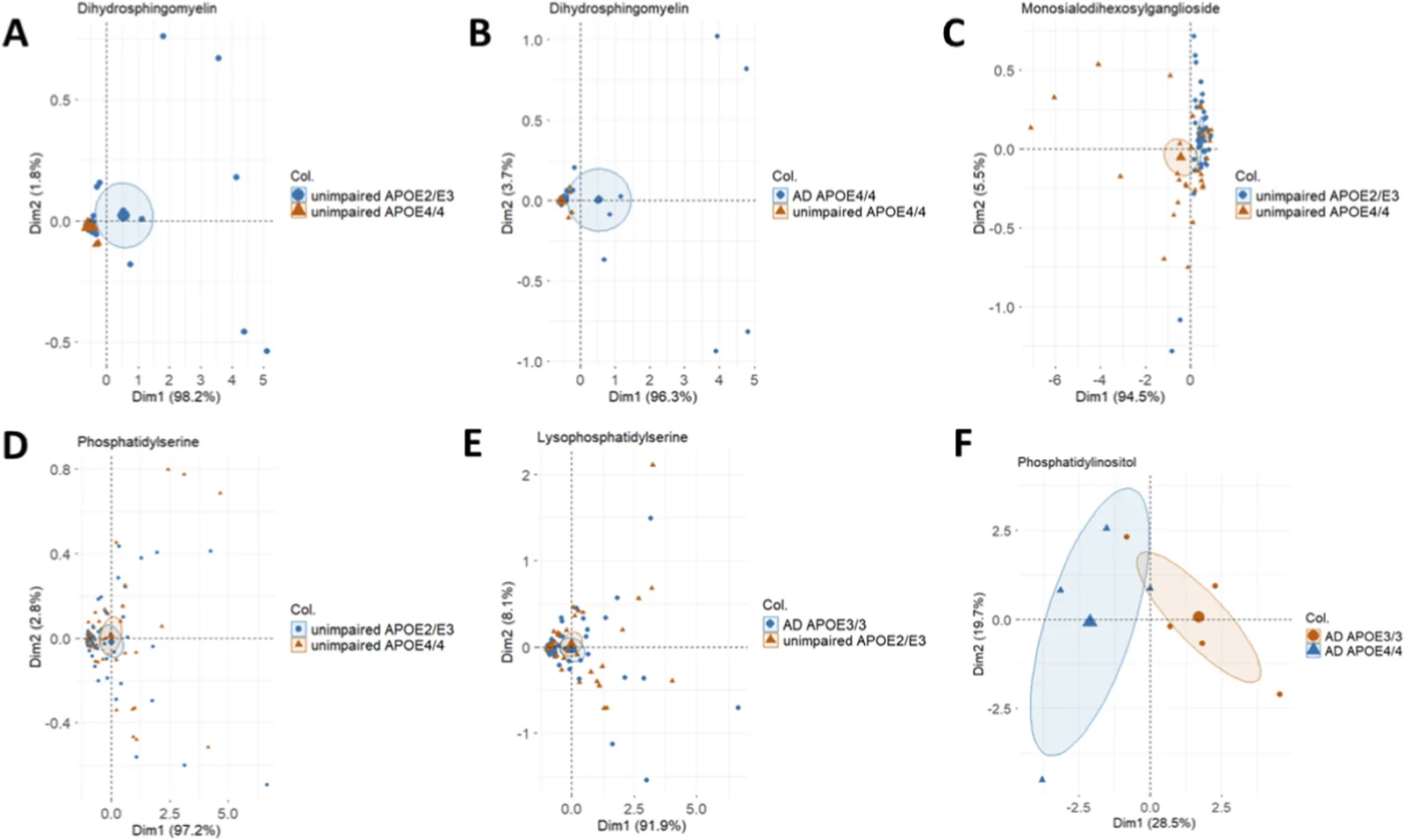
Principal component analysis (PCA) of lipidomic profiles across APOE-genotype groups in EVs from hiPSC-derived neurons of unimpaired control and AD samples. **(A)** Dihydrosphingomyelin (dhSM) genotype Effect: High PC1 (98.2%) shows a clear metabolic separation between unimpaired APOE2/E3 (blue) and unimpaired APOE4/4 (orange) individuals. **(B)** Dihydrosphingomyelin (dhSM) disease effect: Significant variance (PC1: 96.3%) illustrates the AD-driven disruption of lipid stability specifically within APOE4/4 carriers (AD vs. Healthy). **(C)** Monosialodihexosylganglioside (GM3) genotype effect: Dominant PC1 (94.5%) indicates early dysregulation and variance dispersion in unimpaired APOE4/4 carriers compared to non-carriers. **(D)** Phosphatidylserine (PS) genotype effect: Maximum discriminatory power (PC1: 97.2%) reflects a consistent and distinct shift in PS levels between unimpaired APOE2/E3 and unimpaired APOE4/4 samples prior to AD onset. **(E)** Lysophosphatidylserine (LPS) disease effect: High PC1 (98.5%) highlights a distinct metabolic reorganization and pathological shift in AD APOE3/3 patients compared to unimpaired controls. **(F)** Phosphatidylinositol (PI) AD-genotype comparison: Clear directional separation on PC1 (28.5%) between AD APOE4/4 (orange) and AD non-APOE4/4 (blue), showing the combined impact of disease and genotype on the PI species lipidomic fingerprint. The axes represent the first two Principal Components (PC1 and PC2), derived from the analysis of the dataset following auto-scaling (centering and scaling). The percentages indicated next to each axis label denote the proportion of total data variance explained by that specific component. Each data point represents one of the four biological replicates (n = 4) per group, with colors corresponding to genotypes: blue for non-carriers (APOE2/E3 or APOE3/3) and orange for APOE4/4 carriers.

Among glycerophospholipid species, the APOE4 genotype was associated with a dominant metabolic shift in phosphatidylserine (PS) levels in unimpaired individuals (PC1: 97.2%), reflecting an early, systematic reduction in this class (Figure 4D). Phosphatidylserines differentiated samples from APOE3/3 AD donors from those of APOE2/E3 unimpaired donors (Figure 4E). Furthermore, phosphatidylethanolamine (PE) profiles differentiated samples from AD donors from samples of unimpaired controls within the APOE4/4 group, suggesting that a synergy between genetic risk and may be further modulated in the context of AD pathology. Moreover, phosphatidylinositol species differentiated samples from APOE4/4 AD donors from samples from APOE2/E3 AD ones (Figure 4F).

The use of Volcano plots enabled simultaneous assessment of fold-change magnitude and statistical stringency, thereby isolating key lipid species with potential diagnostic or functional significance. In APOE4/4-derived samples, the presence of AD was marked by a systematic depletion of several structural and signaling lipids. Beyond the alterations in ganglioside metabolism (e.g., GM3 d18:1/16:0 and d18:1/18:0) (Figure 5B), the most significant downward shifts were in the SM, PI, and PCe classes. Specifically, dhSM d18:0/18:1 and PI 38:3 emerged as top-ranking molecules due to their high -log10(p-value) and negative log2 fold-change (Figures 5A,D), while PS 40:7 and PS 38:4 indicated high −log10(p-value) and positive log2 fold-change (Figure 5C). Furthermore, the significant upregulation of the glycerolphospholipids PI 38:3 and PI 36:3 suggests disruption of PI-mediated signaling pathways specifically associated with the APOE4 genotype and disease progression (Figure 5D).

**FIGURE 5:**
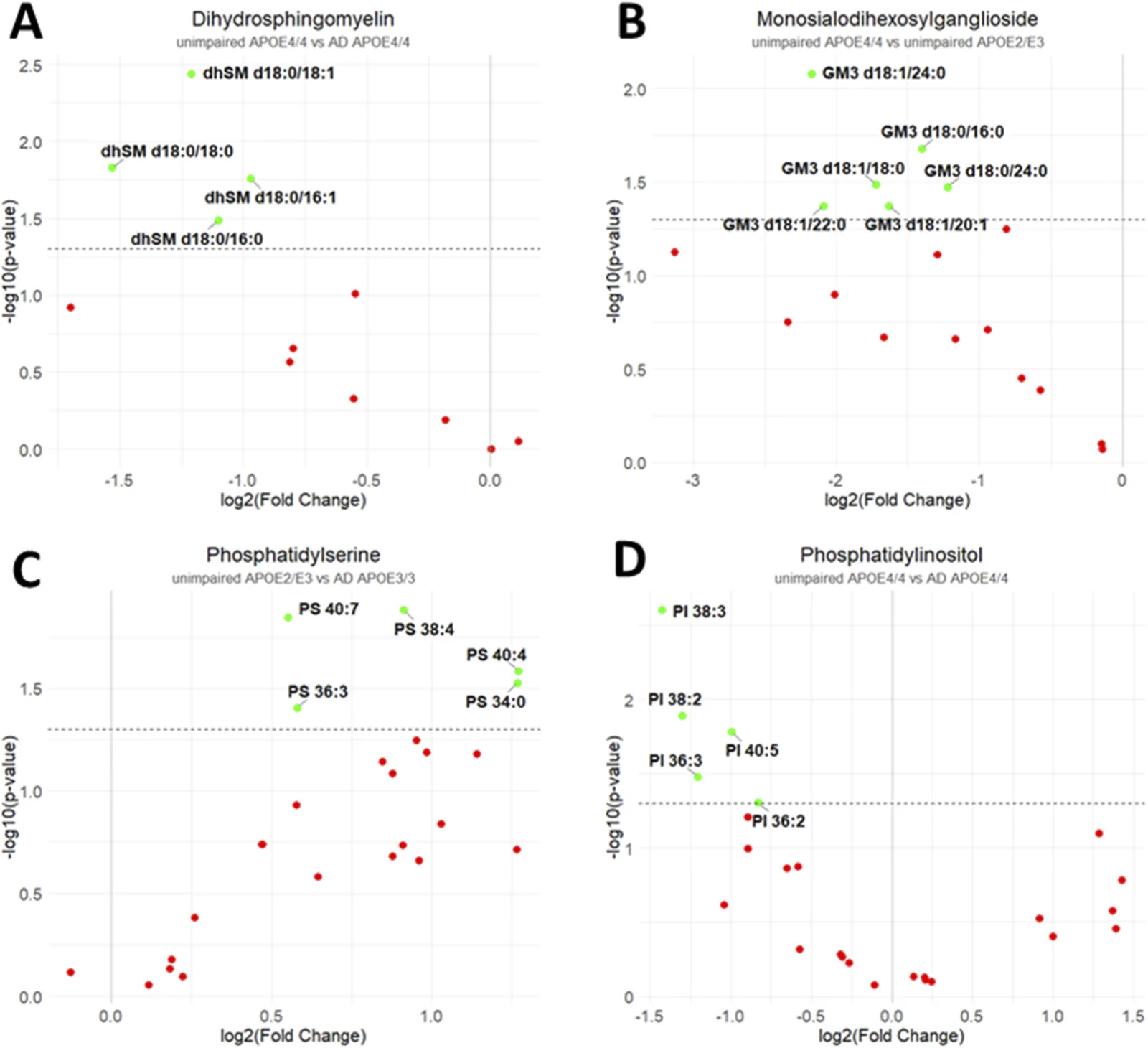
Volcano plots identifying differentially expressed lipid species across EVs from hiPSC-derived neurons (AD and APOE genotypes). **(A)** Dihydrosphingomyelin (dhSM) downregulation in AD APOE4/4. Comparing Healthy APOE4/4 to AD APOE4/4, specific dhSM species crucial for membrane structural integrity and oxidative stress resistance, including dhSM d18:0/18:1, d18:0/18:0, and d18:0/16:1, exhibit significantly reduced levels (log2FC < −1) in the AD group. **(B)** GM3 Ganglioside variation by APOE genotype. Comparison between unimpaired APOE4/4 and unimpaired APOE2/E3 reveals that several GM3 species, such as GM3 d18:1/24:0, d18:0/16:0, and d18:1/18:0, are significantly lower in the APOE4/4 carriers even in the absence of AD, suggesting a genotype-specific baseline lipidomic shift. **(C)** Phosphatidylserine (PS) upregulation in AD APOE3/3. Contrary to other lipids, specific PS species like PS 40:7, 38:4, and 40:4 show a significant increase (log2FC > 0.5) when comparing Healthy APOE2/E3 to AD APOE3/3 patients, potentially reflecting altered apoptotic signaling or synaptic remodeling in non-E4 carriers. **(D)** Phosphatidylinositol (PI) enrichment in AD APOE4/4. Comparison between unimpaired APOE4/4 and AD APOE4/4, with significantly upregulated species PI 38:3 and PI 36:3 in the AD condition. Statistical Note: The plots display the magnitude of change (log2-fold change, x-axis) versus statistical significance (−log10 p-value, y-axis). The dashed horizontal line indicates the significance threshold (p < 0.05), with green points representing lipid species that differ significantly between the groups while red points show non-significant changes. The x-axis represents the magnitude of change (log2 fold-change) and the y-axis indicates statistical significance (−log10 p-value). The dashed horizontal line marks the threshold for statistical significance (p < 0.05). Additionally, fold-change thresholds are indicated (e.g., |log2FC| > 0.5 or 1), with green points highlighting the lipid species that meet both criteria (significance and effect size).

Pairwise comparisons of major lipid classes were performed with multiple-testing correction using the Benjamini–Hochberg False Discovery Rate (FDR) method. Following FDR correction, distinct patterns of significance emerged (Figure 6). Our exploratory analysis identifies that sphingomyelins and dihydrosphingomyelins exhibited a robust disease-driven alteration, with significant differences observed between samples from unimpaired APOE2/E3 and AD APOE4/4 carriers (Figure 6A) as well as between samples from unimpaired APOE4/4 individuals and AD APOE4/4 patients (Figure 6B). Bis(monoacylglycero)phosphates were significantly altered specifically within the APOE4/4 genotype upon disease onset suggesting that AD-associated changes in this lipid class may be influenced by APOE4 status (Figure 6D). Furthermore, sphingomyelins showed significant variance when comparing samples from AD APOE4/4 patients with those from healthy non-carriers, highlighting pronounced metabolic divergence in the diseased at-risk group; however, these differences did not survive FDR correction, suggesting a more subtle effect size of AD for this class. In contrast, phosphatidylinositol displayed a strong genotype-driven difference between unimpaired APOE2/E3 and APOE3/3 individuals as well as unimpaired APOE4/4 carriers and non-carriers, supporting early PI dysregulation, but lost statistical significance in the direct disease comparison after correction (Figure 6C).

**FIGURE 6:**
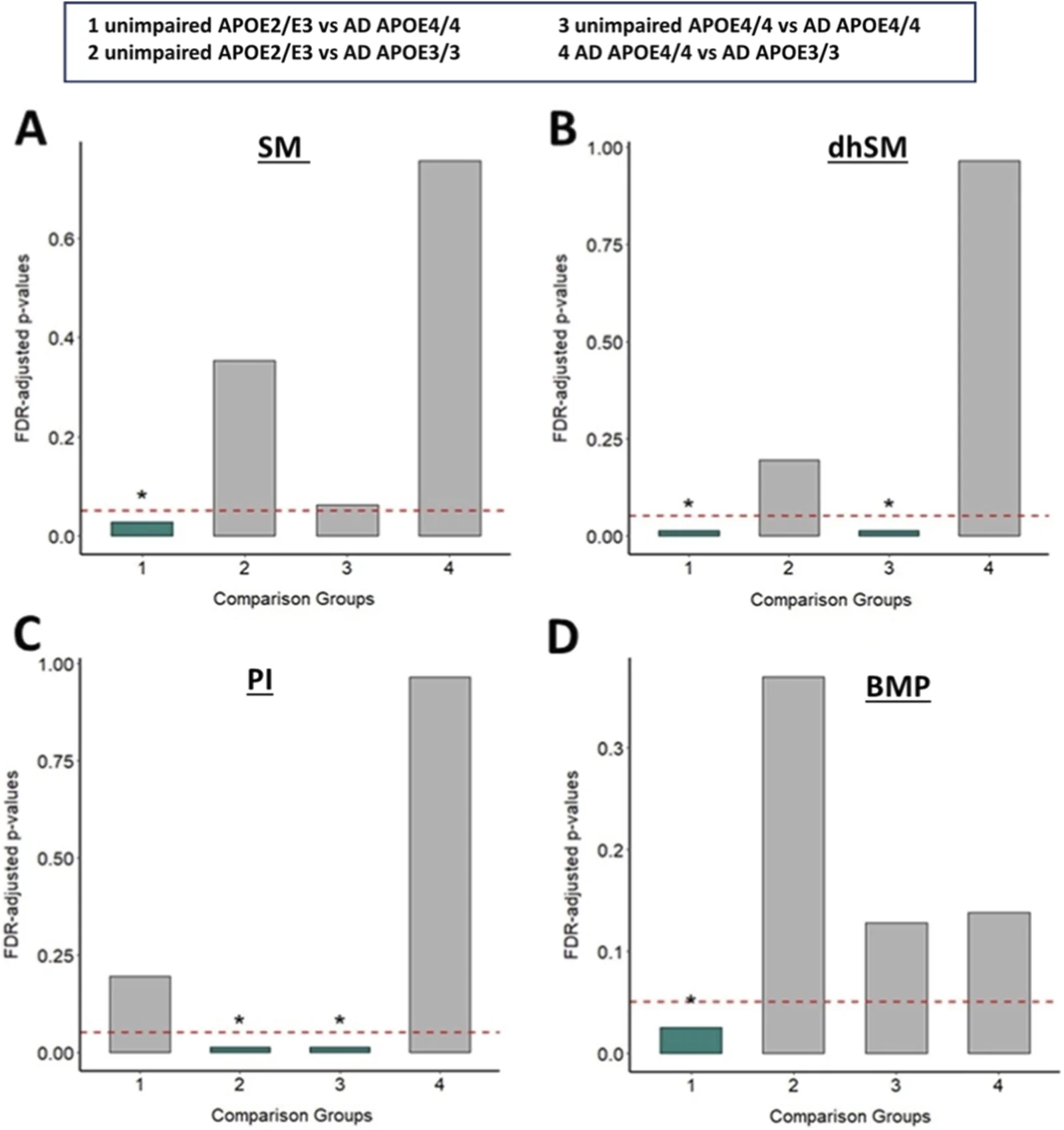
Statistical significance of lipidomic alterations after FDR correction. **(A)** SM; **(B)** dhSM; **(C)** PI; **(D)** BMP. The red dashed line indicates the significance threshold of FDR-adjusted p-value <0.05. Green bars marked with an asterisk (*) represent statistically significant differences between the compared groups, while grey bars indicate non-significant results (Comparisons: 1 is unimpaired APOE2/E3 with AD APOE4/4, 2 is unimpaired APOE2/E3 with AD APOE3/3, 3 is unimpaired APOE4/E4 with AD APOE4/4, 4 is AD APOE4/E4 with AD non-APOE3/3). The analysis was based on four independent biological replicates per group. Due to the high dimensionality of the dataset (593 lipid species), the Benjamini–Hochberg FDR correction was applied to account for multiple testing and control the false discovery rate. The red dashed line represents the stringent statistical significance threshold (p-value <0.05).

## 4 Discussion

The application of EV lipidomics is a promising strategy for investigating AD-related pathogenic processes and for developing clinically useful biomarkers, given the important involvement of lipid metabolism alterations in AD pathophysiology. We performed targeted lipidomic analysis of iPSC derived neuronal EVs to examine the signature of the APOE4/4 genotype and of AD in a controlled human cellular system to help probe molecular signatures linked to genetic susceptibility and/or AD pathophysiology. Given the limited number of independent donors, these findings should be considered exploratory. Significant differences were observed in lipid levels across the SLs, GPLs, and LPLs classes among unimpaired and diseased groups. There appeared to be species-specific lipid alterations that were not apparent at the broader lipid-class level, highlighting the importance of high-resolution lipidomic profiling. Specifically, lipids PC 38:0, PCe 40:7, BMP 38:2, and BMP 40:5 were significantly reduced in EV samples from neurons derived from AD APOE4/4 patients compared with unimpaired APOE4 carriers, suggesting targeted disruptions in pathways associated with membrane integrity and lysosomal metabolism. Multivariate analyses supported these findings, suggesting that specific lipid species disproportionately contributed to group separation and likely reflected underlying processes, such as neuroinflammation, metabolic stress and impaired cellular homeostasis. Collectively, these results suggest that AD in the context of APOE4/4 genotype is characterized by species-specific lipid dysregulation, highlighting the potential for identifying early diagnostic biomarkers and genotype-informed therapeutic strategies (Ma et al., 2025; Van Kruining et al., 2025). However, these findings warrant further investigation in larger cohorts to determine whether these patterns represent a genotype-specific alteration.

Recent iPSCs studies have shown that neuronal differentiation involves significant lipid remodeling, with variations in APOE-related changes across different neural cell types (Odenkirk et al., 2025). In cerebral organoid models, APOE4 was associated with altered cholesterol, glycerophospholipid, and sphingolipid metabolism, whereas APOE deficiency increased fatty-acid utilization and cholesteryl ester accumulation, indicating distinct lipid consequences of APOE4 expression and APOE loss (Zhao et al., 2021; Gadara et al., 2023). Neural/glial based studies have further shown that APOE4-associated cholesteryl ester and lipid-droplet accumulation occurs mainly in astrocytes and microglia rather than neurons (Sienski et al., 2021; Haney et al., 2024; Feringa et al., 2025). In contrast, our analysis of EV secreted from iPSC-derived glutamatergic neurons identified an APOE4-associated, lipid profile involving GM3, PS, PI, dhSM, PC, PCe, and BMP rather than broad neutral-lipid accumulation. Differences in dhSM and GM3 between unimpaired and AD APOE4/4 samples suggest that disease status may further modify the lipidome based on genotype background. Although methodological and biological differences related to the chosen model system limit direct comparisons across studies, distinct lipidomic signatures seem to be associated with both APOE4 and AD.

Ganglioside GM3 levels are increased in the brains of AD patients and in AD rodent models (Dodge et al., 2022). Plasma GM3 levels show a positive association with AD severity in patients, in two large clinical studies (Huynh et al., 2020). Previous work from our group shows enriched levels of GM3 lipid species such as GM3 (d18:0/18:0, d18:0/20:0, d18:1/18:0) in neuronal EVs from AD patients (Krokidis et al., 2024). Gangliosides may facilitate the amyloidogenic processing of amyloid precursor protein, enhancing Aβ aggregation thereby contributing to the formation of amyloid plaques. Binding of Aβ to gangliosides induces a conformational shift from a random coil to a neurotoxic β-sheet structure, which can act as a seed that accelerates amyloid fibril assembly (Choo-Smith et al., 1997; Matsuzaki and Horikiri, 1999). Significantly higher levels of simple gangliosides GM2 and GM3 were detected in the cortex and dentate gyrus of the hippocampus in a APP/PS1 transgenic mouse model of AD proposing that age-related ganglioside dysregulation is spatiotemporally associated with Aβ plaques (Wang et al., 2025). More recent findings indicate that overexpression of *Hexa* and *Hexb,* responsible for the conversion of GM2 to GM3 within lysosomes of microglial cells, results in lysosomal GM3 accumulation, reduced Aβ phagocytosis and enhanced production of proinflammatory cytokines (Park et al., 2026). This reinforces GM3’s role as a candidate biomarker, capable of identifying the AD disease state even within the high-risk genetic background of APOE4/4 carriers, however, further validation in larger, independent cohorts will be required to determine its potential utility as a biomarker for AD disease status.

Volcano plots and FDR-corrected analyses revealed a coordinated reduction in structural, signaling, and protective lipids in the APOE4-associated AD phenotype. Reduced PS levels suggest early neuronal membrane alterations, while decreased PI and SM species in AD point to impaired intracellular signaling and myelin integrity. Furthermore, the pervasive decline of ether-linked PCe species, which serve as essential endogenous antioxidants (Dean and Lodhi, 2018), suggests that AD pathology in the presence of APOE4 leads to a failure of the antioxidant shield. Together, these findings propose a model in which genetic risk and Alzheimer’s disease pathology may act synergistically to shape the lipidome into a distinct molecular profile, with the identified lipid species representing promising candidates for further investigation as potential biomarkers and therapeutic targets in genetically susceptible populations.

Extensive evidence supports a central role for APOE4 in modulating lipid metabolism and AD pathophysiology. Transgenic mouse models expressing human APOE variants have demonstrated that early plasma sphingolipid changes are primarily driven by APOE genotype rather than AD pathology, with APOE4 carriers showing elevated sphingomyelins and reduced ceramides (Bandaru et al., 2009; Van Kruining et al., 2025; Uranbileg et al., 2024). APOE variants directly alter cellular and tissue lipid metabolism, providing a mechanistic basis for their influence on neurodegenerative disease risk and resilience (Yang et al., 2023). Consistent with these observations, EV samples from neurons derived from healthy APOE4 carriers exhibited species-specific alterations in PCs and BMPs, potentially reflecting early-life metabolic advantages, including enhanced membrane function, energy storage, and synaptic efficiency (Patrick, 2019; Oriá et al., 2024). With aging or AD onset, disruption of these lipid pathways leads to oxidative stress, membrane destabilization, and neuroinflammatory activation (Asante et al., 2022; Ebright et al., 2022). This dual effect of APOE4 supports the concept of antagonistic pleiotropy, in which early benefits confer later vulnerability, underscoring the importance of temporal and genotype-specific approaches for understanding AD pathogenesis and identifying early lipid-based biomarkers (Li, 2024; Ma et al., 2025).

Notably, even among healthy individuals, EV samples from neurons derived from APOE4 carriers displayed species-specific lipid alterations, including PC 38:0, PCe 40:7, and BMPs, which were largely absent in unimpaired non-carriers. Thus, APOE4 may selectively modulate lipid composition under physiological conditions, potentially conferring advantages in membrane dynamics, synaptic function, and energy utilization. This enhancement is supported by phosphatidylcholine dynamics that may improve cholinergic neuron function and BMP enrichment that likely boosts endolysosomal activity (Miranda et al., 2022). Choline and choline-containing phospholipids play a critical role in maintaining neurovascular unit integrity and their supplementation particularly with α-glyceryl-phosphorylcholine may support neurovascular unit function and offer therapeutic potential in neurodegenerative and vascular-related brain disorders (Roy et al., 2022). These observations align with previous studies linking species-specific lipid profiles to neuronal resilience, which could later predispose cells to vulnerability in AD (Odenkirk et al., 2024).

In the presence of AD, PC/PCe levels decreased while BMPs increased, indicating impaired membrane homeostasis, lysosomal dysfunction, and elevated inflammatory signaling. Although these findings are derived from human lipid profiles, some have been corroborated in transgenic AD models, showing that early plasma sphingolipid alterations are primarily influenced by APOE genotype rather than overt AD pathology, while neuronal lipid changes are subtle and region-specific (Van Kruining et al., 2025; Uranbileg et al., 2024). Moreover, the accumulation of oxidized lipids and evidence of metabolic exhaustion in AD APOE4 samples further support the transition from protective to deleterious lipid remodeling (Asante et al., 2022).

Our exploratory analysis is consistent with the possibility that the effectiveness of therapeutic interventions targeting lipid metabolism might best be tailored to both age and APOE genotype. While APOE4 may provide early-life metabolic advantages, careful monitoring of lipid profiles and strategies to mitigate oxidative stress may reduce the risk of AD later in life (Al-Ghraiybah et al., 2026). Species-specific lipid alterations in AD APOE4/4 carriers represent promising early risk biomarkers and potential targets for precision therapeutic strategies (Safieh et al., 2019; Yassine et al., 2025). However, given the relatively small number of independent donors included in this study, these observations should be interpreted with caution and require validation in larger, independent cohorts before broader conclusions can be drawn regarding their generalizability to the wider AD population or their potential clinical relevance. Moreover, future research should focus on longitudinal cohort studies to monitor lipid dynamics, functional studies in cellular and animal models to elucidate mechanistic pathways, and assessment of the therapeutic potential of lipid-targeted interventions. Overall, these findings suggest that AD in APOE4/4 carriers is marked by species-specific lipid dysregulation, highlighting the need to integrate peripheral and central lipidomic analyses and reinforcing the potential of genotype-informed interventions and early biomarkers to advance precision medicine approaches.

## 5 Conclusion

The present work presents a lipidomic characterization of neuronal EVs in the context of AD and the APOE genotype, integrating multi-layered statistical analyses to identify lipid alterations consistent with potential biological relevance. By comparing the lipid composition of EVs across four clinically and genetically stratified groups—unimpaired APOE4/4, unimpaired APOE2/E3, AD APOE4/4, and AD APOE3/3—we identified lipid alterations associated with APOE4 status and with the presence of AD. The APOE4 variant may influence lipid composition even in EVs derived from neurons of cognitively unimpaired donors. In EVs derived from neurons of individuals with AD, APOE4/4 appeared to be associated with more selective, species-specific lipid imbalances rather than broad alterations across lipid classes.

Although lipid class-level comparisons revealed no significant differences after FDR correction, analysis at the individual lipid species level identified differential abundances of PC 38:0, PCe 40:7, BMP 38:2, and BMP 40:5, particularly between healthy and AD APOE4/4 donors. Glycerophospholipids and lysophospholipids emerged as the most affected lipid classes, suggesting that specific lipid species, rather than broad class-level alterations, may represent potential indicators of AD-related molecular changes in APOE4/4 carriers. These findings identify candidate lipid signatures associated with APOE4 and AD status; however, further functional studies are required to determine whether these lipid alterations contribute to disease mechanisms or reflect downstream consequences of AD pathology. Validation of these candidate lipid signatures in independent patient-derived cohorts and complementary experimental systems, including primary neuronal systems, animal models and human biofluids, will be essential to determine their reproducibility, mechanistic relevance and potential value as biomarkers.

Nevertheless, these findings should be interpreted with caution in light of the relatively small sample size and require validation in larger, independent cohorts before broader conclusions can be drawn regarding their generalizability to the wider AD population or their potential utility for biomarker development and therapeutic applications.

## Data Availability

Data generated in the present investigation are included in this article. Study-specific data availability statements are available on reasonable request by contacting the corresponding authors. No original code was developed for this study. The R scripts used for the analyses and visualizations is available at: https://github.com/MariaChristinaPapatheodorou/APOE4-alters-lipid-composition-in-extracellular-vesicles-from-hiPSC-derived-AD-neurons.
