# Supplementary material for: APOE4 differentially alters lipid composition in extracellular vesicles from hiPSC-derived neurons of Alzheimer’s disease patients
Source: Front Pharmacol. 2026 Sep 25;17:1917428. doi: 10.3389/fphar.2026.1917428 (PMC13649956; doi:10.3389/fphar.2026.1917428)
Supplement: Supplementary file 1 [file DataSheet1.docx]

Supplementary Material

Supplementary Table 1: Relative quantitative percentage (%T-lip) of the average of main lipid categories (sterol lipids, glycerolipids, sphingolipids glycerophosholipids and lysophosholipids) obtained from distinct groups of EVs from hiPSC-derived neurons of unimpaired control and AD samples (both APOE2/E3 and APOE4/4)^1^.

| Lipid Categories | Unimpaired control APOE2/E3 | Unimpaired control APOE4/4 | AD APOE3/3 | AD APOE4/4 |
| --- | --- | --- | --- | --- |
| Sterol lipids^2^ | 33.62 ± 2.59* | 28.81 ± 2.55 | 30.46 ± 3.39 | 28.12 ± 5.50 |
| Glycerolipids^3^ | 11.83 ± 2.11 | 11.08 ± 2.12 | 13.42 ± 3.39 | 22.02 ± 10.02 |
| Sphingolipids^4^ | 7.68 ± 2.49^&^ | 7.60 ± 1.17 | 7.38 ± 1.32 | 6.56 ± 1.89 |
| Glycerophospholipids^5^ | 50.39 ± 1.30^#^ | 44.44 ± 6.90^##^ | 45.95 ± 3.04 | 40.12 ± 6.90 |
| Lysophospholipids^6^ | 2.15 ± 0.32^&&^ | 1.90 ± 064 | 2.49 ± 0.13 | 2.80 ± 0.67 |

^1^The values are given as mean ± SD (n = 4). ^2^sterol lipids include FC+CE; ^3^glycerolipids are MG+DG+TG; ^4^sphingolipids are CEr+dhCer+SM+dhSM+MhCer+sulf+LacCer+GM3+GB3; ^5^glycerophospholipids are PA+PC+PCe+PE+PEp+PS+PI+ PG+BMP; ^6^lysophospholipids are LPC+LPE+LPI+LPS. Values for each lipid class are presented in Table S1; Statistically significant samples p < 0.05, p < 0.01. *For sterols (unimpaired control APOE2/E3 vs. unimpaired control APOE4/4; p < 0.05), ^&^for sphingolipids (unimpaired control APOE2/E3 vs. unimpaired control APOE4/4; p < 0.05), ^#^for glycerophospholipids (unimpaired control APOE2/E3 vs. unimpaired control APOE4/4; p < 0.05), ^#^for glycerophospholipids (unimpaired control APOE4/4 vs. AD APOE4/4; p < 0.05), ^&&^for lysophospholipids (unimpaired control APOE2/E3 vs. AD APOE3/3).

Supplementary Table 2: The *p* values of the average of main lipid categories (sterol lipids, glycerolipids, sphingolipids and glycerophosholipids) obtained from distinct groups of EVs from hiPSC-derived neurons of unimpaired control and AD samples (both APOE2/E3 and APOE4/4).

| Lipid Categories | Unimpaired control APOE2/E3 vs. Unimpaired control APOE4/4 | Unimpaired control APOE2/E3 vs. AD APOE3/3 | Unimpaired control APOE4/4 vs. AD APOE4/4 | AD APOE4/4 vs. AD APOE3/3 |
| --- | --- | --- | --- | --- |
| Sterol lipids | 0.0332 | 0.10061 | 0.42481 | 0.25452 |
| Glycerolipids | 0.33812 | 0.31013 | 0.09028 | 0.15143 |
| Sphingolipids | 0.04977 | 0.14779 | 1.08067 | 0.29839 |
| Glycerophospholipids | 0.04383 | 0.34492 | 0.0479 | 0.18255 |
| Lysophospholipids | 0.30315 | 0.02827 | 2.35363 | 0.21395 |

This table presents the complete statistical results. All comparisons are based on n = 4 biological replicates per group. Statistical significance was evaluated using unpaired two-tailed Student's t-tests (or non-parametric tests where required by the data distribution). To control for multiple testing across the 593 lipid species, adjusted p-values (q-values) were calculated using the Benjamini–Hochberg False Discovery Rate (FDR). Effect sizes are reported as log2 fold-changes log2 FC), with the observed changes ranging from approximately 0.6- to 2.0-fold (d ≈ 0.04–1.9), a range consistent with the biological variability of neuronal EVs.

The two raw p-values (1.08067 and 2.35363) exceeding 1.0 were identified as data entry or export errors and have been verified against the original statistical output. Across the lipid class comparisons, fold changes ranged from 0.6 to 2.0, corresponding to effect sizes (Cohen's d) ranging from small to large (|d| ≈ 0.04–1.9), based on n = 4 biological replicates per group.

Following Benjamini–Hochberg correction, none of the nominally significant differences (raw p < 0.05) remained significant at the false discovery rate (FDR) threshold (q < 0.05), indicating that these lipid class-level differences should be interpreted as exploratory findings rather than statistically significant.

Supplementary Table 3: Relative quantitative percentage (%T-lip) of the average of main lipid clases obtained from distinct groups of EVs from hiPSC-derived neurons of unimpaired control and AD samples (both APOE2/E3 and APOE4/4)^1^.

| Lipid Classes | Unimpaired control APOE2/E3 | Unimpaired control APOE4/4 | AD APOE3/3 | AD APOE4/4 |
| --- | --- | --- | --- | --- |
| FC-%T-lip | 31.60±2.38 | 26.91 ± 2.95 | 28.37 ± 3.81 | 24.72 ± 6.67 |
| CE-%T-lip | 2.05 ± 0.32 | 1.91 ± 0.46 | 2.09 ± 0.46 | 3.40 ± 1.27 |
| MG-%T-lip | 10.73 ± 1.84 | 10.02 ± 1.94 | 12.22 ± 3.41 | 20.14 ± 9.38 |
| DG-%T-lip | 0.58 ± 0.12 | 0.57 ± 0.12 | 0.65 ± 0.17 | 0.91 ± 0.37 |
| TG-%T-lip | 0.52 ± 0.19 | 0.49 ± 0.08 | 0.55 ± 0.12 | 0.97 ± 0.36 |
| dhCer-%T-lip | 0.02 ± 0.00 | 0.03 ± 0.00 | 0.03 ± 0.01 | 0.04 ± 0.01 |
| Cer-%T-lip | 0.76 ± 0.09 | 0.79 ± 0.03 | 0.88 ± 0.22 | 0.95 ± 0.22 |
| SM-%T-lip | 2.06 ± 0.38 | 2.43 ± 0.37 | 2.20 ± 0.67 | 1.30 ± 0.19 |
| dhSM-%T-lip | 1.38 ± 0.17 | 1.68 ± 0.25 | 1.39 ± 0.49 | 0.85 ± 0.14 |
| MhCer-%T-lip | 0.87 ± 0.20 | 0.73 ± 0.16 | 0.78 ± 0.20 | 1.17 ± 0.50 |
| Sulf-%T-lip | 2.16 ± 1.82 | 1.07 ± 0.29 | 1.31 ± 0.26 | 1.80 ± 0.93 |
| LacCer-%T-lip | 0.08 ± 0.01 | 0.10 ± 0.03 | 0.12 ± 0.04 | 0.12 ± 0.03 |
| GM3-%T-lip | 0.28 ± 0.04 | 0.66 ± 0.17 | 0.42 ± 0.12 | 0.24 ± 0.09 |
| GB3-%T-lip | 0.07 ± 0.01 | 0.12 ± 0.04 | 0.24 ± 0.10 | 0.10 ± 0.03 |
| PA-%T-lip | 0.32 ± 0.06 | 0.32 ± 0.02 | 0.35 ± 0.02 | 0.37 ± 0.06 |
| PC-%T-lip | 23.46 ± 2.90 | 25.87 ± 4.20 | 22.33 ± 3.12 | 16.03 ± 6.80 |
| PCe-%T-lip | 1.03 ± 0.27 | 1.13 ± 0.16 | 0.84 ± 0.22 | 0.74 ± 0.02 |
| PE-%T-lip | 7.70 ± 1.23 | 8.50 ± 1.98 | 8.66 ± 1.23 | 9.93 ± 1.65 |
| PEp-%T-lip | 6.67 ± 1.11 | 7.26 ± 1.19 | 6.42 ± 0.91 | 8.43 ± 2.42 |
| PS-%T-lip | 2.58 ± 0.74 | 3.68 ± 0.58 | 4.54 ± 1.05 | 2.28 ± 0.95 |
| PI-%T-lip | 2.46 ± 0.16 | 3.35 ± 0.39 | 2.57 ± 0.44 | 2.12 ± 0.56 |
| PG-%T-lip | 0.18 ± 0.05 | 0.22 ± 0.03 | 0.21 ± 0.03 | 0.20 ± 0.03 |
| BMP-%T-lip | 0.05 ± 0.01 | 0.06 ± 0.02 | 0.04 ± 0.01 | 0.03 ± 0.00 |
| LPC-%T-lip | 0.34 ± 0.10 | 0.26 ± 0.08 | 0.28 ± 0.02 | 0.35 ± 0.08 |
| LPCe-%T-lip | 0.01 ± 0.01 | 0.01 ± 0.00 | 0.01 ± 0.00 | 0.01 ± 0.00 |
| LPE-%T-lip | 0.77 ± 0.21 | 0.82 ± 0.35 | 1.10 ± 0.27 | 1.11 ± 0.20 |
| LPEp-%T-lip | 0.59 ± 0.07 | 0.46 ± 0.15 | 0.63 ± 0.21 | 0.82 ± 0.34 |
| LPI-%T-lip | 0.28 ± 0.08 | 0.19 ± 0.08 | 0.24 ± 0.04 | 0.32 ± 0.09 |
| LPS-%T-lip | 0.16 ± 0.05 | 0.16 ± 0.08 | 0.24 ± 0.04 | 0.19 ± 0.03 |

^1^The values are given as mean ± SD (n = 4).

Αll values are presented as mean ± SD, based on n = 4 independent biological replicates per group, and are expressed as normalized relative abundance (mol%). Although the sample size was limited, the tight clustering observed in the principal component analysis suggests that the lipidomic profiles primarily reflect biological variation rather than technical noise.

Supplementary Table 4: The *p* values of the average of lipid classes obtained from distinct groups of of EVs from hiPSC-derived neurons of unimpaired control and AD samples (both APOE2/E3 and APOE4/4).

| Lipid Families | Unimpaired control APOE2/E3 vs. Unimpaired control APOE4/4 | Unimpaired control APOE2/E3 vs. AD APOE3/3 | Unimpaired control APOE4/4 vs. AD APOE4/4 | AD APOE4/4 vs. AD APOE3/3 |
| --- | --- | --- | --- | --- |
| FC-%T-lip | 0.0449 | 0.0642 | 0.3186 | 0.2153 |
| CE-%T-lip | 0.3369 | 0.4641 | 0.0871 | 0.1191 |
| MG-%T-lip | 0.3321 | 0.3027 | 0.0919 | 0.153 |
| DG-%T-lip | 0.4294 | 0.2289 | 0.1286 | 0.1995 |
| TG-%T-lip | 0.3971 | 0.439 | 0.0605 | 0.1133 |
| dhCer-%T-lip | 0.0473 | 0.1639 | 0.1768 | 0.2832 |
| Cer-%T-lip | 0.3058 | 0.2282 | 0.1534 | 0.3764 |
| SM-%T-lip | 0.0802 | 0.3879 | 0.0137 | 0.0679 |
| dhSM-%T-lip | 0.0487 | 0.4845 | 0.0135 | 0.0902 |
| MhCer-%T-lip | 0.2425 | 0.3426 | 0.125 | 0.1496 |
| Sulf-%T-lip | 0.2278 | 0.2254 | 0.222 | 0.2292 |
| LacCer-%T-lip | 0.1811 | 0.0752 | 0.3405 | 0.2527 |
| GM3-%T-lip | 0.0121 | 0.0566 | 0.0114 | 0.0397 |
| GB3-%T-lip | 0.0531 | 0.0329 | 0.155 | 0.0437 |
| PA-%T-lip | 0.4917 | 0.2676 | 0.1513 | 0.3661 |
| PC-%T-lip | 0.2731 | 0.3788 | 0.0495 | 0.1287 |
| PCe-%T-lip | 0.238 | 0.1979 | 0.0149 | 0.249 |
| PE-%T-lip | 0.3052 | 0.0195 | 0.0089 | 0.2205 |
| PEp-%T-lip | 0.3146 | 0.3755 | 0.1949 | 0.1063 |
| PS-%T-lip | 0.0043 | 0.0374 | 0.0603 | 0.0654 |
| PI-%T-lip | 0.0139 | 0.3277 | 0.0082 | 0.0347 |
| PG-%T-lip | 0.0729 | 0.1452 | 0.1555 | 0.3447 |
| BMP-%T-lip | 0.1425 | 0.0467 | 0.0312 | 0.1315 |
| LPC-%T-lip | 0.2138 | 0.1901 | 0.1249 | 0.1081 |
| LPCe-%T-lip | 0.1796 | 0.2046 | 0.1004 | 0.1584 |
| LPE-%T-lip | 0.4006 | 0.1434 | 0.1185 | 0.4554 |
| LPEp-%T-lip | 0.1258 | 0.3964 | 0.0672 | 0.2817 |
| LPI-%T-lip | 0.1435 | 0.2747 | 0.2285 | 0.202 |
| LPS-%T-lip | 0.4988 | 0.0213 | 0.2847 | 0.011 |

The table provides the complete statistical results for the lipid class comparisons. Statistical significance was assessed using unpaired two-tailed Student's t-tests. Due to the high dimensionality of the dataset (593 lipid species), all p-values were adjusted for multiple testing using the Benjamini–Hochberg false discovery rate (FDR) procedure. Effect sizes are reported as log2 fold changes (log2FC), allowing direct assessment of the magnitude and direction of lipid abundance changes. The volcano plots (Figure 5) illustrate these directional changes together with the corresponding statistical significance metrics.

**Supplementary Figure 1.** Relative quantitative percentage of representative classes of A) free cholesterol, B) dihydroceramide, C) phosphatidylcholine, D) ether-linked phosphatidylcholine, E) Bis(monoacylglycerol)phosphate, F) lysophosphatidylserine. The values are given as mean ± SD (n = 4) in EVs from hiPSC-derived neurons of unimpaired control and AD samples (both APOE2/E3 and APOE4/4). Statistical significance: * (p < 0.05).

**Supplementary Figure 2.** PCA of lipidomic profiles across APOE-genotype groups in EVs from hiPSC-derived neurons of unimpaired control and AD samples. A) Monosialodihexosylganglioside AD APOE3/3 compared to AD APOE4/4, B) Monosialodihexosylganglioside AD APOE3/3 compared to unimpaired APOE2/E3, C) Phosphatidylethanolamine AD APOE4/4 compared to unimpaired APOE4/4, D) Phosphatidylinositol unimpaired APOE2/E3 compared to unimpaired APOE4/4, E) Bis(monoacylglycerol)phosphate AD APOE4/4 compared to unimpaired APOE4/4, F) Lysophosphatidylserine AD APOE3/3 compared to AD APOE4/4. The axes represent the first two Principal Components (PC1 and PC2), derived from the analysis of the dataset following auto-scaling (centering and scaling). The percentages indicated next to each axis label denote the proportion of total data variance explained by that specific component. Each data point represents one of the four biological replicates (n=4) per group, with colors corresponding to genotypes: blue for non-carriers (APOE2/E3 or APOE3/3) and orange for APOE4/4 carriers.

**Supplementary Figure 3.** Volcano plots identifying differentially expressed lipid species across EVs from hiPSC-derived neurons (AD and APOE genotypes). A) GM3, comparing unimpaired APOE4/4 and AD APOE4/4. B) GM3, comparing AD APOE4/4 to AD APOE4/4, C) Gobotriaosylceramide, comparing unimpaired APOE2/E3 and AD APOE3/3, D) Ether-linked phosphatidylcholine, comparing unimpaired APOE4/4 and AD APOE4/4, E) Phoshpatodylinositol, comparing unimpaired APOE4/4 and AD APOE4/4, F) is(monoacylglycerol)phosphate, comparing unimpaired APOE4/4 and AD APOE4/4. The x-axis represents the magnitude of change (log2 fold-change) and the y-axis indicates statistical significance (-log10 p-value). The dashed horizontal line marks the threshold for statistical significance (p < 0.05). Additionally, fold-change thresholds are indicated (e.g., |log2FC| > 0.5 or 1), with green points highlighting the lipid species that meet both criteria (significance and effect size).

**Supplementary Figure 4.** t-SNE analysis of lipidomic profiles between APOE genotype and disease status groups in extracellular vesicles (EVs) derived from hiPSC-neurons, corresponding to Figure 4. t-SNE was applied to the same lipid class datasets as in Figure 4. (A) Genotype effect on dihydrosphingomyelin (dhSM): unimpaired APOE2/E3 (blue) and unimpaired APOE4/4 (orange) form distinct, non-overlapping clusters, consistent with the separation shown by PCA. (B) Disease effect on dihydrosphingomyelin (dhSM): AD APOE4/4 (blue) and unimpaired APOE4/4 (orange) samples separate clearly along tSNE-1, mirroring the disease-induced disruption observed with PCA. (C) Genotype effect on monosialoganglioside (GM3): unimpaired APOE2/E3 (blue) and unimpaired APOE4/4 (orange) show partial overlap, indicating a less robust separation than suggested by PCA. (D) Genotype effect on phosphatidylserine (PS): unimpaired APOE2/E3 (blue) and unimpaired APOE4/4 (orange) form clearly separated clusters, in line with PCA findings. (E) Disease effect on lysophosphatidylserine (LPS): AD APOE3/3 (blue) and unimpaired APOE2/E3 (orange) samples exhibit substantial overlap, suggesting that the PCA-derived separation for this comparison should be interpreted with caution. (F) AD-genotype comparison for phosphatidylinositol (PI): AD APOE3/3 (orange) and AD APOE4/4 (blue) occupy distinct regions of the projection, consistent with the separation shown by PCA.

**Supplementary Figure 5.** t-SNE analysis of lipidomic profiles across additional APOE-genotype and disease group comparisons, corresponding to Supplementary Figure 2. (A) Monosialodihexosylganglioside (GM3) AD-genotype comparison: AD APOE3/3 (orange) and AD APOE4/4 (blue) show partial overlap, with a diagonal trend distinguishing the two groups. (B) Monosialodihexosylganglioside (GM3) disease effect: AD APOE3/3 (blue) and unimpaired APOE2/E4 (orange) samples show moderate overlap. (C) Phosphatidylethanolamine (PE) disease effect: AD APOE4/4 (blue) and unimpaired APOE4/4 (orange) form clearly separated, non-overlapping clusters. (D) Phosphatidylinositol (PI) genotype effect: unimpaired APOE2/E3 (blue) and unimpaired APOE4/4 (orange) show substantial overlap. (E) Bis(monoacylglycero)phosphate (BMP) disease effect: AD APOE4/4 (blue) and unimpaired APOE4/4 (orange) show partial separation with some overlap. (F) Lysophosphatidylserine (LPS) AD-genotype comparison: AD APOE3/3 (orange) and AD APOE4/4 (blue) form separate clusters, though based on a very limited number of samples.
